# Evidence of facultative parthenogenesis in three Neotropical pitviper species of the *Bothrops atrox* group

**DOI:** 10.7717/peerj.10097

**Published:** 2020-11-18

**Authors:** Sergio D. Cubides-Cubillos, José S.L. Patané, Karina Maria Pereira da Silva, Selma Maria Almeida-Santos, Denise S. Polydoro, Guilherme Guidolin Galassi, Silvia Regina Travaglia Cardoso, Maria José de J. Silva

**Affiliations:** 1 Laboratório de Ecologia e Evolução, Instituto Butantan, São Paulo, Brazil; 2 Laboratório Especial de Ciclo Celular, Instituto Butantan, São Paulo, Brazil; 3 Aquário Municipal de Campinas, Campinas, São Paulo, Brazil; 4 Parque Ecológico Municipal de Americana, Americana, São Paulo, Brazil; 5 Museu Biológico, Instituto Butantan, São Paulo, Brazil

**Keywords:** Serpentes, Squamata, Neotropical snakes, *B. moojeni*, *B. leucurus*, Automixis, Genetic markers, Microsatellites

## Abstract

We examined four suspected cases of facultative parthenogenesis in three species of a neotropical lineage of pitvipers of the *Bothrops atrox* group. Reproduction without mating was observed in captive females of *B. atrox, B. moojeni* and* B. leucurus* housed alone for seven years (the two former species) and nine years (the latter one). In addition to the observation of captivity data, we investigated molecularly this phenomenon using heterologous microsatellites. DNA was extracted from the mothers’ scales or liver, from embryo and newborn fragments, and yolked ova. Four of the microsatellites showed good amplification using Polymerase Chain Reaction and informative band segregation patterns among each mother and respective offspring. Captivity information, litter characteristics (comparison of the number of newborns, embryos and yolked ova) and molecular data altogether agreed with facultative parthenogenesis predictions in at least three out of the four mothers studied: *B. atrox* (ID#933) was heterozygous for three out of the four markers, and the sons S1 and S2 were homozygous; *B. moojeni* (BUT86) was heterozygous for two out of four markers, offspring S1, S3, E2, and E4, and O1 to O6 were homozygous; and *B. leucurus* (MJJS503) was heterozygous for three out of four markers, and son E1 and O1 were homozygous. *B. moojeni* (BUT44) was homozygous for all loci analyzed in the mother and offspring, which although not informative is also consistent with parthenogenesis. This study represents the first molecular confirmation of different pitviper species undergoing facultative parthenogenesis among Neotropical endemic snakes.

## Introduction

Parthenogenesis (virgin birth) *sensu lato* has been defined as a mode of asexual reproduction (Vrijenhoek, 1999; Avise, 2008). True parthenogenesis is sperm-independent production of offspring, in contrast to other unisexual reproductive modes, such as gynogenesis and hybridogenesis, in which sperm is needed at some level (Neaves & Baumann, 2011). Obligate parthenogenesis (OP) is relatively more common in plants and invertebrates (Bell, 1982), occurring only in some reptilian lineages within vertebrates (Kearney, Fujita & Ridenour, 2009). The only known obligate parthenogenetic snake lineage is the Brahminy blind snake, *Indotyphlops braminus*, previously known as *Ramphotyphlops braminus* (McDowell, 1974; Nussbaum, 1980; Wynn, Cole & Gardner, 1987; Ota et al., 1991).

Switching between sexual and asexual reproduction is called facultative parthenogenesis (FP), and it was first reported in turkey and chicken (Olsen, 1975; Neaves & Baumann, 2011). Several cases of facultative parthenogenesis in vertebrates have been described considering the last twenty years or so, suggesting that the detection of this phenomenon can increase if more species are investigated. Nowadays, it is known to occur in a number of vertebrate species from different lineages (Kearney, Fujita & Ridenour, 2009): elasmobranch fishes (e.g., Chapman et al., 2007; Chapman, Firchau & Shivji, 2008; Feldheim et al., 2010; Robinson et al., 2011; Portnoy et al., 2014; Fields et al., 2015; Harmon et al., 2015; Dudgeon et al., 2017; Feldheim et al., 2017; Straube et al., 2016), lizards (e.g., Lenk et al., 2005; Watts et al., 2006; Lampert, 2008; Hennessy, 2010; Wiechmann, 2012; Grabbe & Koch, 2014; Miller et al., 2019), birds (Olsen, 1967; Olsen, 1970; Olsen, 1975; Schut, Hemmings & Birkhead, 2008; Parker & McDaniel, 2009; reviewed in Ramachandran & McDaniel, 2018), and snakes (Booth & Schuett, 2016; Shibata et al., 2017; Allen, Sanders & Thomson, 2018; Seixas et al., 2020).

Although facultative parthenogenesis had been suggested as potentially adaptive, facilitating the establishment of a population prior to the introduction of genetically diverse conspecifics (Hedrick, 2007), some authors hypothesized facultative parthenogenesis as a consequence of reproductive error and/or a side-effect of isolation from males (Avise, 2008; Lampert, 2008). More recently, however, cases of facultative parthenogenesis in wild populations of snakes, described in *Agkistrodon contortrix* and *A. piscivorus* (Booth & Schuett, 2011; Booth et al., 2012), ruled out the hypothesis that only captive females could undergo this phenomenon (Booth & Schuett, 2016).

Facultative parthenogenesis induces elevated homozygosity, depending on the exact mechanism, possibly precluding its persistence for long evolutionary periods (Hedrick, 2007), and even though facultative parthenogenesis increases the risk for lower fitness (Hedrick, 2007; Kearney, Fujita & Ridenour, 2009), some authors suggest that this feature may have an important evolutionary role in purging deleterious alleles, therefore diminishing the population’s genetic load (Hedrick, 1994; Crnokrak & Barrett, 2002).

### Parthenogenesis in *Bothrops*

*Bothrops* is a Neotropical endemic genus (Martins, Marques & Sazima, 2002; and references therein), viviparous, which produces litters of two to 86 offspring in the summer and autumn (Almeida-Santos & Salomão, 2002; Barros, Rojas & Almeida-Santos, 2014; Silva et al., 2019).

Phylogenetic studies performed by Fenwick et al. (2009), Carrasco et al. (2012) and Alencar, Quental & Grazziotin (2016) showed that *B. atrox*, *B. moojeni*, and *B. leucurus* belong to the *atrox* group. *Bothrops atrox* occurs throughout most of the northern part of South America (Wüster, Thorpe & Puorto, 1996; Campbell & Lamar, 2004; Nogueira et al., 2019), *B. moojeni* occupies central and southeastern Brazil and adjacent Paraguay and Argentina (Campbell & Lamar, 2004; Nogueira et al., 2019), and *B. leucurus* is mainly distributed throughout northeastern Brazil (Carvalho Jr & Nascimento, 2005; Lira-da Silva, 2009; Nogueira et al., 2019).

Batistic et al. (1999), Almeida-Santos & Salomão (2002) and Vaughan & Steele (2014), based on captivity information, suggested that parthenogenesis had occurred in *B. moojeni, B. insularis*, and *B. asper*, respectively. Nevertheless, the phenomenon was not confirmed with molecular data in any of the cases.

In this study, we employed molecular markers (microsatellites) to investigate the hypothesis of facultative parthenogenesis in four suspected cases of Neotropical pitvipers genus *Bothrops*: *B. atrox* (one specimen), *B. moojeni* (two specimens), and *B. leucurus* (one specimen), altogether with captivity information available for each species analyzed genetically.

## Material and Methods

### Specimens studied and captivity history

#### Bothrops atrox

The *B. atrox* female (ID #933) was born in Rondônia state, Brazil and was formerly housed in Morungaba (São Paulo state, Brazil) since its birth in 2006. It was subsequently held in isolation until it was transferred to the Criadouro Conservacionista de Americana, where it was again housed in isolation. In April 2010, the mother gave birth to a fully developed and apparently normal neonate, a malformed embryo, and five yolked ova. The neonate died in June 2010. Almost one year later, in March 2011, this female gave birth to a neonate (born dead) and ten yolked ova. In March 2013, the female gave birth to a living male with malformation and 23 yolked ova (Table 1).

**Table 1:** Data on reproductive females of the genus *Bothrops* that may indicate facultative parthenogenesis. Included (+)/Not included (-) means that the samples were or not included in the present study.

**Species Mother (Mo)**	**Lab no.**	**Mother’s birth**	**Offspring birth date**	**Offspring**		**Embryos**	**Yolked Ova Expelled (O)**	**Sex in cluth**	**Included/ Not included**
				**Alive**	Dead				
*B. atrox*	ID#933	2006	2010 (April)	1 (S1)	–	1	5	Male	+
			2011 (March)		1	–	10	?	–
			2013 (March)	1 (S2)		–		Male	+
*B. moojeni*	BUT44	2006	2010 (January)	6*	1^⧫^		21	–	–
			2011 (March)	3	1	–	–	?	+
			2013 (March)	4		–	8	Males	+
*B. moojeni*	BUT86	2006	2015 (July, Oct.)	–		–	24	–	–
			2016 (Oct.)	1 (S1)	–	1 (E2)*	39 (O1–O3)	Male	+
			2018 (Sept.)	1 (S3)		1 (E4)	31 (O4–O6)	Male	+
*B. leucurus*	MJJS503	2010	2019/(Feb.)	–		1 (E1)*	31 (O1)	?	+

**Notes.**

Rhombus (⧫) indicates a partially formed individual.

Asterisks (*) indicate (i) fully-formed individuals with abnormalities (and after the birth, the mother BUT44 ate the offspring) and (ii) sex was not detectable in embryos.

Question mark (?) indicates that the sex was not defined because (i) the mother ate the offspring, or (ii) the sons were malformed and composed of an amorphous mass in the terminal region of the body, or (iii) the animals died during the weekend and they were found deteriorated physically on Monday.

#### Bothrops moojeni

Two independent cases were observed in *B. moojeni*: the first female (BUT44) was born in captivity in 2006 at the Sorocaba Zoo (Sorocaba, São Paulo state) and donated to the Aquário Municipal de Campinas (Campinas, São Paulo state) while still a newborn; and the animal has been held at that location ever since. In January 2010, the female gave birth to five neonates (fully formed with minor abnormalities), twenty-one expelled yolked ova and one partially formed embryo (Fig. S1). In March 2011, the same isolated female had three more offspring, of which one died and two survived. In March 2013, the female gave birth to four dead offspring and eight unpreserved yolked ova. We analyzed molecularly the mother and three offspring (Table 1).

The second *B. moojeni* female (BUT86) was born in the Museu Biológico, Instituto Butantan, in December 2006; and in August 2013, it was donated to the Laboratório de Ecologia e Evolução at the same Institute. In July 2015, the first litter of this mother encompassed 24 yolked ova. In October 2015, another yolked ovum was found (we did not have access to these samples for genetic study). In October 2016, the female gave birth to an alleged living offspring, an embryo, and 39 yolked ova. In September 2018, it gave birth to a neonate (who died about 3 h after birth), an embryo, and 31 yolked ova. We analyzed molecularly the mother, one neonate, an embryo, and three ova from 2016; and one neonate, an embryo, and three ova from 2018 (Table 1).

#### Bothrops leucurus

The *B. leucurus* female (MJJS503) arrived at the Museu Biológico, Instituto Butantan, as a young specimen in March 2010 and was isolated from males for several years. In February 2019, it gave birth to a malformed neonate (embryo) and 31 yolked ova. The mother, the embryo and the content of one ovum were analyzed using molecular markers (Table 1).

In some cases, the sex of descendants from the respective putative parthenogenetic mother was not detected because: (i) the mother ate the offspring, or (ii) the descendants were malformed and composed of an amorphous mass in the terminal region of the body, or (iii) the animals were born during the weekend, and they were found dead and disintegrated on the next Monday (Table 1).

All individuals studied herein were approved for the Ethics Committee for the Use of Animals of Instituto Butantan (CEUAIB) under the approval numbers 14/1260 and 2141020819.

### Methods

In the new four suspected cases reported herein, the presence of testes in stillborn and viable offspring was observed (when possible) by microscopy. For the molecular analyses, DNA was extracted from scales, embryo fragments and yolked ova using a modified Chelex©100 (BioRad) protocol (Walsh, Metzger & Higuchi, 1991). Proteinase K (20 mg/mL—Thermo Fisher) was added for tissue digestion at 56 °C overnight (24, 48 and 74 h). Polymerase chain reactions (PCR) were carried out in a LifeECO—Bier thermocycler, using nine heterologous microsatellite primers developed for *Agkistrodon contortrix* (Castoe et al., 2010), *Bothrops marmoratus* (Machado, 2015) and *B. insularis* (K. Zamudio, pers. comm., 2013) from which only four amplified (Table 2). PCR were performed in final volumes of 15 µL with the following specifications: 2,625 µL H_2_O, 1.5 µL 10 × Buffer, 0.60 µL MgCl_2_ (50 mM-Invitrogen), 0.3 µL dNTP (5 mM-Invitrogen), 1.2 µL primer forward (2.5 µM), 1.2 µL primer reverse (2.5 µM), 0.075 µL *Platinum* Taq polymerase (Invitrogen), and 7.5 µL of DNA (30 ng/µL). PCR conditions for all the primers consisted of denaturation at 95 °C for 5 min., followed by 35 cycles of (i) denaturation at 94 °C for 1 min.; (ii) annealing varied from 56 to 62 °C (Table 2) for 1 min.; and (iii) extension at 72 °C for 1 min., with a final extension at 72 °C for 5 min. PCR products and a molecular weight standard (Low Mass Ladder and 1 kb—Life Technologies) were loaded into individual wells of a 2.2 or 2.5% agarose gel prepared with 1x TBE and Gel Red (Biotium). Amplified DNA was subsequently run at 85 volts in an electrophoresis apparatus for 70 min (Bi 52.13 and Bi 60.3 microsatellites) and 90 min (Ac4335 and MR102 microsatellites), using the same buffer used for gel preparation. The results were visualized under UV light, and the images saved as digital pictures.

**Table 2:** Microsatellite primers and respective species from which they were obtained, used for amplifying sequences of the mothers and the respective offspring for the suspected parthenogenesis cases in *Bothrops atrox*, *B. moojeni*, and *B. leucurus*.

**Locus**	**Species**	**DNA strand**	**Primer sequences**	**Annealing** (T °C)	**References**
**Microsatellites**					
Ac4335	*Agkistrodon contortrix*	5′	ATC CTT CCC CAA GCC AAG G	62	Castoe et al. (2010)
		3′	GCT GGA GAC TGG AGA AGA GAG C		
MR102	*Bothrops marmoratus*	5′	CTC TTT TGC AGT TAT GGC CC	56	Machado (2015)
		3′	TGG CTT AGG AAG ACA CTG AAA		
Bi52.13	*B. insularis*	5′	TAC TGT ATT GCA CCG GCT AAG G	62; 56^a^	K Zamudio (pers. comm., 2013)
		3′	AAT CTC CTG TTT TAA TGC TAC TGA A		
Bi60.3	*B. insularis*	5′	CTT TGC CGC CGA TGG TG	60	K Zamudio (pers. comm., 2013)
		3′	GGT TGG GCC TGT GGA CTG TT		

**Notes.**

a Annealing temperature varied according to the species: 62 °C for *B. moojeni* and 56 °C for *B. leucurus*.

The sizes of amplified bands for each individual were obtained using GelQuant.NET v1.7.8 (biochemlabsolutions.com), by comparing to the DNA bands of the molecular weight standard as depicted above.

## Results

Nine loci (Ac4335, MR102, Bi60.3, Bi60.6, Bi52.7, Bi52.8, Bi52.13, Bi52.17, Bi52.22) were tested, however only Ac4335, MR102, Bi52.13, and Bi60.3 generated gel patterns with bands segregating between mother and offspring (Table S1).

**Figure 1:**
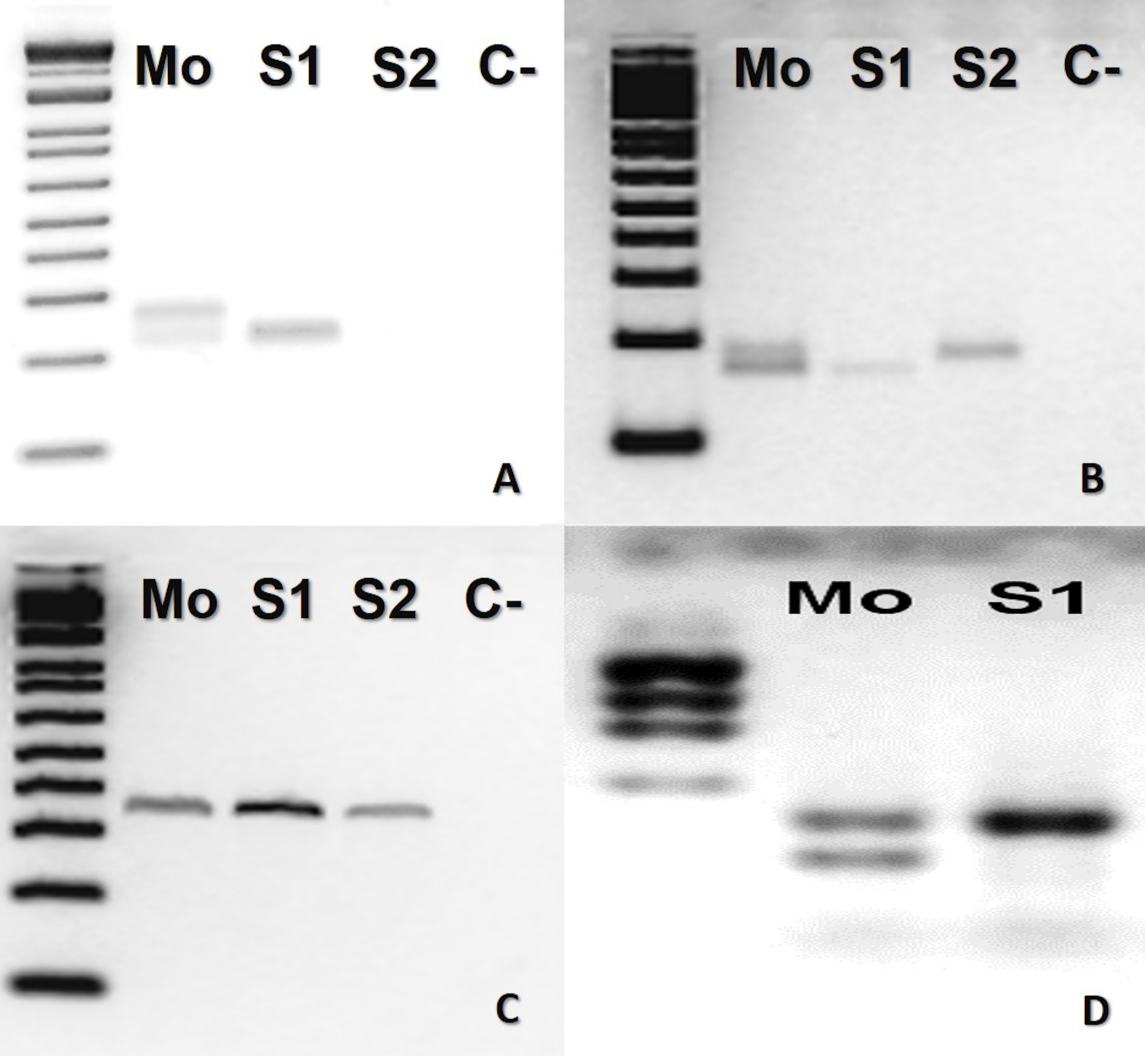
*Bothrops atrox* (ID# 933) PCR microsatellite bands in electrophoretic agarose gels. (A–D), where Mo, putative parthenogenetic mother; S1 and S2, sons of Mo; (C-), negative control. The first lane in all figures is a molecular weight standard. Loci: (A) Ac4335, (B) MR102, and (C) Bi52.13 run with Low Mass Ladder 1kb and (D) Bi60.3 with Low Mass Ladder 2 kb.

In *B. atrox*, four loci (Ac4335, MR102, Bi52.13, and Bi60.3) were informative (Fig. 1). The mother was heterozygous for three loci (Ac4335, MR102, Bi60.3), whilst the offspring was homozygous (Figs. 1A, 1B and 1D). For locus Bi52.13, the mother and the descendants S1 and S2 shared the same band (Fig. 1C). We could not obtain results using the marker Bi60.3 for the second son (S2).

**Figure 2:**
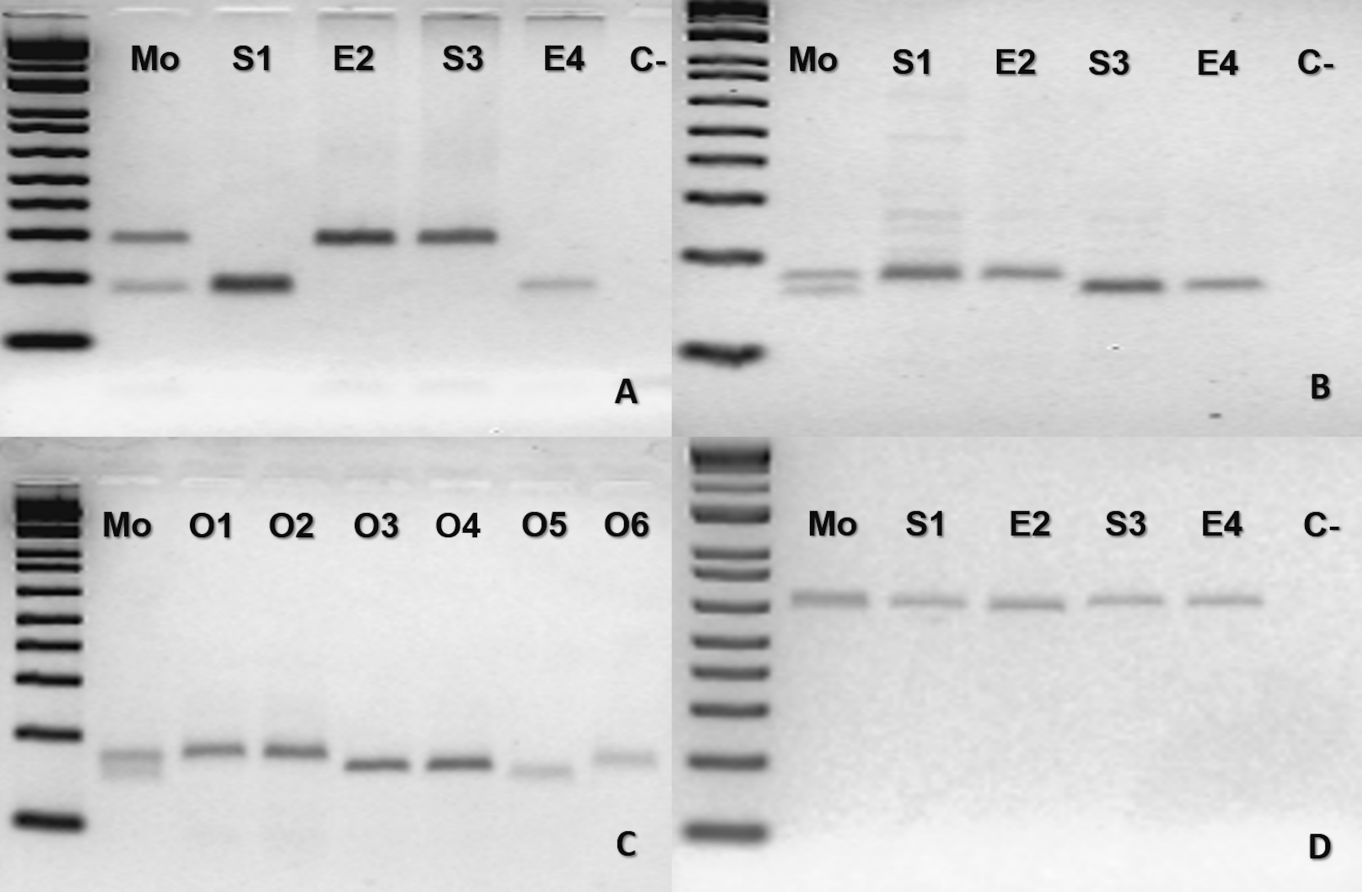
*Bothrops moojeni* (BUT86) PCR molecular marker bands in electrophoretic agarose gels. (A–D), where Mo, putative parthenogenetic mother; S1, E2, S3 and S4, sons of Mo (S1 and E2 were born in 2016, and S3 and E4 were born in 2018); O1–O6: ova (O1–O3 from 2016 litter, and O4–O6 from 2018 litter); (C-): negative control. The first lane in all figures is a molecular weight standard. Loci: (A) Ac4335. (B–C) MR102. (D) Bi52.13; all the markers were run together with Low Mass Ladder 1 kb.

The mother *B. moojeni* (BUT44) and its offspring from different litters shared the same homozygous band for the loci tested MR102 and Bi52.13 (Fig. S2) being, therefore, uninformative for testing facultative parthenogenesis. For the second case of *B. moojeni* (BUT86), the mother was heterozygous for two loci (Ac4335 and MR102) and the offspring was homozygous (Figs. 2A, 2B and 2C). Each litter, from 2016 and 2018, showed individuals with different bands for the locus Ac4335, with each band being shared with the mother (Fig. 2A). Regarding locus MR102, the 2016 litter (which includes the descendants S1 and E2—Fig. 2B, and three ova: O1, O2 and O3—Fig. 2C) and 2018 litter (which includes the descendants S3 and E4—Fig. 2B, and three ova: O4, O5 and O6—Fig. 2C)—the same band was observed in four of the five individuals from the 2016 litter; likewise, O3 shared the same band as four out of the five individuals (S3, E4, O4, and O5) from the 2018 litter (Figs. 2B–2C), while an ovum (O6) was also different from the rest of its generation. Regarding locus Bi52.13, the mother and offspring shared the same band (Fig. 2D). The locus Bi60.3 evinced the same homozygous band being shared by mother and offspring (Fig. S3).

**Figure 3:**
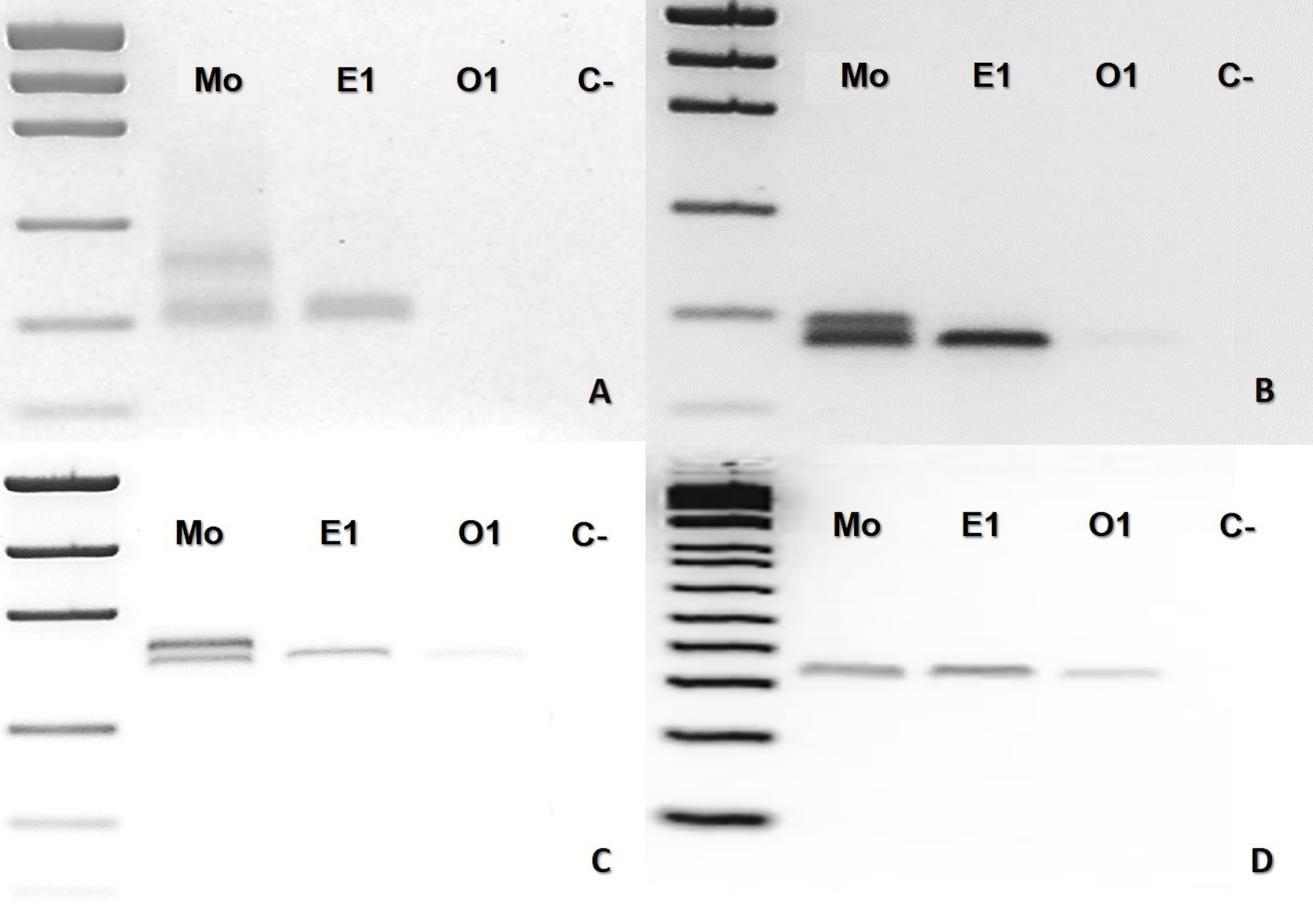
*Bothrops leucurus* (MJJS503) PCR molecular marker bands in electrophoretic agarose gels. (A–D), being Mo, putative parthenogenetic mother; E1, son (embryo) of Mo; O1, one of the yolked ova; (C-), negative control. The first lane in all figures is a molecular weight standard. Loci: (A) Ac4335, (B) MR102 and (C) Bi52.13 with Low Mass Ladder 2kb, and (D) Bi60.3 with Low Mass Ladder 1 kb.

In the case of *B. leucurus,* the mother was heterozygous for three markers (Ac4335, MR102, Bi52.13) and homozygous for marker Bi60.3; the son (E1) was homozygous sharing one band with the mother for each of the four markers (Figs. 3A–3D). An ovum was homozygous for the same band of the son for loci MR102, Bi52.13, and Bi60.3, which were also shared with the mother (Figs. 3B, 3C and 3D).

## Discussion

Parthenogenesis, initially conceived as most common in plants and invertebrates, has been increasingly detected within vertebrates (Booth & Schuett, 2016; Ramachandran & McDaniel, 2018). In some of these cases, the offspring present delayed and unorganized development (Ramachandran & McDaniel, 2018).

In the present study, considering both captivity and molecular data altogether, the results confirmed for the first time facultative parthenogenesis in an endemic Neotropical genus of pitvipers (*Bothrops*), specifically in three species of the same clade (*B. atrox* group). Thus, molecular data supported the assumption that *B. atrox* (ID #933), *B. moojeni* (BUT86), and *B. leucurus* (MJJS503) offspring were born as the result of facultative parthenogenesis since the offspring showed homozygosity for heterozygous loci in the mother (Figs. 1A, 1B, 1D, 2A, 2B and 3A–3C).

Furthermore, other features concerning the offspring were also found here: (i) none of the mothers had been housed with males (ruling out events of long-term sperm storage), (ii) there was a relatively large number of undeveloped ova, and (iii) only alleles present in the mother (either in homozygosity or heterozygosity) were observed in the offspring.

After considering those points, it is also worth highlighting that captivity data of all four cases approached herein agreed with the facultative parthenogenesis predictions outlined by Booth & Schuett (2011), given that the two amplified loci were found homomorphic in the mother *B. moojeni* (BUT44) and its sons. So, although there was no definitive molecular support for facultative parthenogenesis in this single case, the absence of additional alleles in the offspring, as well as the fact that the mother did not have contact with males since its birth are in agreement with the facultative parthenogenesis hypothesis.

In addition and assuming that each mother in the four cases analyzed herein came from a Mendelian population in Hardy-Weinberg equilibrium, as reported by Shibata et al. (2017), the relationship between the number of alleles and the number of haplotypes could help to estimate the allelic frequency for each marker. Then, after calculating the probability of obtaining the same offspring in a normal process of sexual reproduction, the hypotheses of paternity or long-term storage of sperm can be rejected, thus confirming the parthenogenesis for the cases reported herein. This scenario is supported by the low combined probability of the genotypes observed in the offspring, evinced by the following values: 1.9E−3 observed for the *B. atrox* (ID #933) case, 2.2E−10 for *B. moojeni* (BUT86) case, and 3.0E−5 for *B. leucurus* (MJJS503) case.

Regarding *B. moojeni* (BUT86), different cells had fused during the meiotic process, since we observed different bands in the same litter with two markers (Figs. 2A and 2B).

Following Stenberg & Saura (2009), the possible cytogenetic mechanisms of facultative parthenogenesis are compiled in Table S2. In the apomixis process, or mitotic parthenogenesis, diploid (2n) eggs are produced because the oocyte undergoes a single maturation division, almost indistinguishable from mitosis. Contrarily, automictic parthenogenesis (automixis), which is based on meiosis, can be subdivided into mechanisms that cause loss of heterozygosity (gamete duplication, terminal fusion, central fusion, and random fusion) and mechanisms in which the genotype of the mother is passed to the offspring without changes (premeiotic doubling and gonoid thelytoky). In that aspect, Booth & Schuett (2016, and references therein) showed that terminal fusion automixis (TFA) is the most common mode of facultative parthenogenesis in snakes, after analyzing different cases reported in the literature. Thus, in terminal fusion automixis, the litter is composed solely of males due to homozigosity of the sex chromosomes in “advanced” (Caenophidia) snakes (ZZ = viable males; WW = unviable; (Booth & Schuett, 2016) and references therein), whilst in more “primitive” lineages (Alethinophidia) the sex determination is of the XX/XY type (XX = viable females; YY = unviable) in at least some lineages (Gamble et al., 2017).

The karyotype for the mother *B. moojeni* (BUT44) showed 2*n* = 36, being 16 macrochromosomes (including the sex pair, ZW) and 20 microchromosomes (Senzaki, 2020), exactly as those reported for other *Bothrops* (Beçak & Beçak, 1969), evincing that no significant divergence in chromosomal morphologies were detected, at least in this case. As observed in birds and some lizards (e.g., lacertids and varanids), some parthenogenetic lineages of Caenophidian snakes exhibit females as the heterogametic sex (ZW) and males as the homogametic sex (ZZ), and in this case only males are produced in the offspring (Olsen, 1975; Watts et al., 2006; Pokorná et al., 2014; Rovatsos et al., 2015). On the other hand, some species of constricting snakes (e.g., *Boa imperator* and *Python bivittatus*)—with XX/XY sex determination-system and males heterogametic (except for *Acrantophis dumerili* that has a ZZ/ZW determination-system)—offspring is composed of females (Gamble et al., 2017). In fact, it has been suggested that males and females heterogametic in snakes, has evolved independently at least two times among the phylogenetically distinct heterogametic lineages (Augstenová et al., 2018). Indeed, our data agree with this hypothesis, since exclusively males were observed (in all the cases we were able to define the sex of the offspring), and these data fit in the type B facultative parthenogenesis, according to Booth & Schuett (2016).

Besides, in the three species studied herein (based on molecular information), previous studies based exclusively on captivity information have already suggested the occurrence of facultative parthenogenesis in *B. moojeni* (Batistic et al., 1999) and *B. insularis*, although long-term sperm storage could not be ruled out (Almeida-Santos & Salomão, 2002). In fact, *B. insularis* is a close relative of *B. jararaca* (Fenwick et al., 2009; Carrasco et al., 2012), a species for which meiosis abnormalities, such as chromosome doubling in oogonia and male aneuploid gametes are known (Beçak, Beçak & Pereira, 2003). This case indicates that the induction of facultative parthenogenesis by such meiotic event may not be an unusual outcome. More recently, facultative parthenogenesis was also observed in *Bothrops asper* by Vaughan & Steele (2014), again without molecular support. Still, according to Almeida-Santos & Salomão (2002), long-term sperm storage is known to occur in different *Bothrops* species, raising the possibility that these two reproductive modes can both occur in the genus. It may be that there is a correlation between characterization of facultative parthenogenesis and long-term sperm storage in many snake lineages, due to the putative erroneous interpretation of undetected occurrence of mating (in captivity or in the wild) as facultative parthenogenesis, when they are in fact a long-term sperm storage situation (e.g., Schuett, 1992; Siegel & Sever, 2006; Smith et al., 2009; Smith, Schuett & Schwenk, 2010; Hoss et al., 2011). However, we agree with Booth & Schuett (2011) that previous reports of long-term sperm storage may have been overestimated, as the number of cases of facultative parthenogenesis keeps increasing, suggesting the latter may indeed be more common than previously detected.

Adding the new results reported herein to those available in the literature, facultative parthenogenesis attested by molecular markers and/or captivity data has been detected in a total of 27 species: *Crotalus horridus*, *C. unicolor*, *C. viridis*, *Agkistrodon contortrix*, *A. piscivorus*, *Bothrops asper, B. atrox, B. insularis, B. moojeni, B. leucurus* (Viperidae), *Oxyuranus scutellatus, Acanthophis antarticus* (Elapidae), *Boa constrictor*, *Epicrates maurus*, *E. cenchria*, *Eunectes murinus, Chilabothrus angulifer* (Boidae), *Python bivittatus*, *P. regius*, *P. brongersmai*, *Malopython reticulatus* (Pythonidae), *Acrochordus arafurae* (Acrochordidae), *Thamnophis elegans vagrans*, *T. marcianus*, *T. radix*, *T. couchii*, and *Nerodia sipedon* (Colubridae) (Batistic et al., 1999; Almeida-Santos & Salomão, 2002; Booth et al., 2011a; Booth et al., 2011b; Vaughan & Steele, 2014; revision in Booth & Schuett, 2016; Shibata et al., 2017; Allen, Sanders & Thomson, 2018; Seixas et al., 2020).

Additionally, as new cases of parthenogenesis have increased, new perspectives on integrative researches have also been emerging; for instance, Calvete et al. (2018) investigated the composition and function of the venom of one male—resulting from automictic parthenogenesis of a mother—of *Agkistrodon contortrix* and two unrelated wild representatives in order to study the consequences of loss of genetic variability in the parthenogenetic male. The results evinced high level of similarity between the venom of the mother and that one of the parthenogenetic offspring, despite the loss of overall allelic diversity in the latter.

It is worth reinforcing the importance of new studies to investigate the possibility of facultative parthenogenesis as a more pragmatic evolutionary process in New World vipers.

## Conclusions

Three cases of facultative parthenogenesis in the Neotropical pitviper genus *Bothrops* were confirmed by molecular markers (heterologous microsatellites) and captivity information altogether. Infertile eggs or non-viable ova and malformed offspring showed to be also very common in those cases. Besides, these are the first cases with molecular evidence in the literature regarding Neotropical pitvipers, so it is possible that further cases in different related species reveal that such a trace may be present as an ancient characteristic in other New World pitviper lineages as well.

Future multidisciplinary studies involving molecular testing, ecological, and evolutionary approaches may shed a light on the putative correlation and effects of different modes of reproduction.

##  Supplemental Information

10.7717/peerj.10097/supp-1 Figure S1 B. moojeni. (A) Mother BUT44. (B) Offspring of mother BUT44. (C) Malformed embryo—attached to de yolk—of mother BUT44. (D) Hemipenis of the embryo of mother BUT44.

10.7717/peerj.10097/supp-2 Figure S2 *Bothrops moojeni* (BUT44) PCR molecular marker bands in electrophoretic agarose gels (A–B), where Mo: putative parthenogenetic mother; S1–S3; (C-): negative control. The first lane in all figures is a molecular weight standard. Loci: (A) MR102. (C) Bi52.13.

10.7717/peerj.10097/supp-3 Figure S3 *Bothrops moojeni* (BUT86) PCR molecular marker bands in electrophoretic agarose gel for locus Bi60.3 Where Mo: putative parthenogenetic mother; S1, E2, S3, and S4: sons of Mo (S1 and E2 were born in 2016, and S3 and E4 were born in 2018) and (C-): negative control. In the first lane: the molecular Low Mass Ladder 1kb.

10.7717/peerj.10097/supp-4 Table S1 Genotypes of mothers and offspring *Loci* from which informative PCR bands of three out of four suspected *Bothrops* facultative parthenogenesis cases were obtained, depicted by species. Numbers refer to estimated gel band sizes.

10.7717/peerj.10097/supp-5 Table S2 Known cytogenetic mechanisms involved in animal parthenogenesis (compiled from (Stenberg & Saura, 2009)) “Autosome composition” assumes that the mother is heterozygous in a specific locus. Crossing-over, as depicted here, is between the centromere and the locus.

## Additional Information and Declarations

### Data Availability

The following information was supplied regarding data availability: The present study uses only band fragment sizes of a few markers, gel images, and specimen images available in the Supplemental Files.

## References

[ref-1] Alencar LRV, Quental TB, Grazziotin F (2016). Diversification in vipers: phylogenetic relationships, time of divergence and shifts in speciation rates. Molecular Phylogenetics and Evolution.

[ref-2] Allen L, Sanders KL, Thomson VA (2018). Molecular evidence for the first records of facultative parthenogenesis in elapid snakes. Royal Society Open Science.

[ref-3] Almeida-Santos SM, Salomão MG, Schuett GW, Hoggren M, Douglas ME, Greene HW (2002). Reproduction in Neotropical pitvipers, with emphasis on species of the genus *Bothrops*. Biology of the Vipers.

[ref-4] Augstenová B, Mazzoleni S, Kratochvíl L, Rovatsos M (2018). Evolutionary dynamics of the W chromosome in caenophidian snakes. Gene.

[ref-5] Avise JC (2008). Clonality: the genetics, ecology, and evolution of sexual abstinence in vertebrate animals.

[ref-6] Batistic RF, Federsoni PA, Calixto SC, Vitiello N (1999). Partenogênese facultative em *Bothrops moojeni*: um caso. V congresso latinoamericano de herpetologia, 1999.

[ref-7] Barros VA, Rojas CA,  Almeida-Santos SM (2014). Reproductive biology of Bothrops erythromelas from the Brazilian Caatinga. Advances in Zoology.

[ref-8] Beçak W, Beçak ML (1969). Cytotaxonomy and chromosomal evolution in Serpentes. Cytogenetics.

[ref-9] Beçak ML, Beçak W, Pereira A (2003). Somatic pairing, endomitosis and chromosome aberrations in snakes (Viperidae and Colubridae). Anais da Academia Brasileira de Ciências.

[ref-10] Bell G (1982). The masterpiece of nature: the evolution and genetics of sexuality.

[ref-11] Booth W, Johnson DH, Moore S, Schal C, Vargo EL (2011a). Evidence for viable, non-clonal but fatherless *Boa* constrictors. Biology Letters.

[ref-12] Booth W, Million L, Reynolds RG, Burghardt GM, Vargo EL, Schal C, Tzika AC, Schuett GW (2011b). Consecutive virgin births in the New World boid snake, the Colombian rainbow boa, Epicrates maurus. Journal of Heredity.

[ref-13] Booth W, Schuett GW (2011). Molecular genetic evidence for alternative reproductive strategies in North American pitvipers (Serpentes, Viperidae): long-term sperm storage and facultative parthenogenesis. Biological Journal of the Linnean Society.

[ref-14] Booth W, Schuett GW (2016). The emerging phylogenetic pattern of parthenogenesis in snakes. Biological Journal of the Linnean Society.

[ref-15] Booth W, Smith CF, Eskridge PH, Hoss SK, Mendelson 3rd JR, Schuett GW (2012). Facultative parthenogenesis discovered in wild vertebrates. Biology Letters.

[ref-16] Calvete JJ, Casewell NR, Hernández-Guzmán U, Quesada-Bernat S, Sanz L, Rokyta DR, Booth W (2018). Venom Complexity in a pitviper produced by facultative parthenogenesis. Scientific Reports.

[ref-17] Campbell JA, Lamar WW (2004). The venomous reptiles of the Western Hemisphere.

[ref-18] Carrasco PA, Mattoni CI, Leynaud GC, Scrocchi GJ (2012). Morphology, phylogeny and taxonomy of South American bothropoid pitvipers (Serpentes, Viperidae). Zoologica Scripta.

[ref-19] Carvalho Jr RR, Nascimento LB (2005). *Bothrops leucurus* geographical distribution. Herpetological Review.

[ref-20] Castoe TA, Poole AW, Gu W, De Koning APJ, Daza JM, Smith EN, Pollock DD (2010). Rapid identification of thousands of copperhead snake (*Agkistrodon contortrix*) microsatellite loci from modest amounts of 454 shotgun genome sequence. Molecular Ecology Resources.

[ref-21] Chapman DD, Firchau B, Shivji MS (2008). Parthenogenesis in a large-bodied requiem shark, the blacktip *Carcharhinus limbatus*. Journal of Fish Biology.

[ref-22] Chapman DD, Shivji MS, Louis E, Sommer J, Fletcher H, Prodöhl PA (2007). Virgin birth in a hammerhead shark. Biology Letters.

[ref-23] Crnokrak P, Barrett SCH (2002). Purging the genetic load: a review of the experimental evidence. Evolution.

[ref-24] Dudgeon CL, Coulton L, Bone R, Ovenden JR, Thomas S (2017). Switch from sexual to parthenogenetic reproduction in a zebra shark. Scientific Reports.

[ref-25] Feldheim KA, Chapman DD, Sweet D, Fitzpatrick S, Prodöhl PA, Shivji MS, Snowden B (2010). Shark virgin birth produces multiple, viable offspring. Journal of Heredity.

[ref-26] Feldheim KA, Clews A, Henningsen A, Todorov L, McDermott C, Meyers M, Bradley J, Pulver A, Anderson E, Marshall A (2017). Multiple births by a captive swellshark *Cephaloscyllium ventriosum* via facultative parthenogenesis. Journal of Fish Biology.

[ref-27] Fenwick AM, Gutberlet Jr RL, Evans JA, Parkinson CL (2009). Morphological and molecular evidence for phylogeny and classification of South American pitvipers, genera *Bothrops*, *Bothriopsis*, and *Bothrocophias* (Serpentes: Viperidae). Zoological Journal of the Linnean Society.

[ref-28] Fields AT, Feldheim KA, Poulakis GR, Chapman DD (2015). Facultative parthenogenesis in a critically endangered wild vertebrate. Current Biology.

[ref-29] Gamble T, Castoe TA, Nielsen SV, Schield DR, Schuett GW, Booth W (2017). The discovery of XY sex chromosomes in a *Boa* and *Python*. Current Biology.

[ref-30] Grabbe J, Koch A (2014). First and repeated cases of parthenogenesis in the varanid subgenus *Euprepiosaurus* (*Varanus indicus* species group) and the first successful breeding of *V. rainerguentheri* in captivity. Biawak.

[ref-31] Harmon TS, Kamerman TY, Corwin AL, Sellas AB (2015). Consecutive parthenogenetic births in a spotted eagle ray *Aetobatus narinari*. Journal of Fish Biology.

[ref-32] Hedrick PW (1994). Purging inbreeding depression. Heredity.

[ref-33] Hedrick PW (2007). Virgin birth, genetic variation and inbreeding. Biology Letters.

[ref-34] Hennessy J (2010). Parthenogenesis in an ornate Nile monitor, *Varanus ornatus*. Biawak.

[ref-35] Hoss SK, Schuett GW, Earley RL, Smith LL (2011). Reproduction in male *Crotalus adamanteus* Beauvois (Eastern diamond-backed rattlesnake): relationship of plasma testosterone to testis and kidney dimensions and mating season. Southeastern Naturalist.

[ref-36] Kearney M, Fujita MK, Ridenour J, Schön I, Martens K, Van Dijk P (2009). Lost sex in the reptiles: constraints and correlations. Lost sex: the evolutionary biology of parthenogenesis.

[ref-37] Lampert KP (2008). Facultative parthenogenesis in vertebrates: reproductive error or chance?. Sexual Development.

[ref-38] Lenk PW, Eidenmueller B, Stauder H, Wicker R, Wink M (2005). A parthenogenetic *Varanus*. Amphibia-Reptilia.

[ref-39] Lira-da Silva RM (2009). *Bothrops leucurus* Wagler, 1824 (Serpentes;Viperidae): natural history, venom and envenomation. Gazeta Médica da Bahia.

[ref-40] Machado T (2015). Filogenia e Filogeografia do grupo *Bothrops neuwiedi* (Serpentes, Squamatas). Ph.D. Thesis.

[ref-41] Martins M, Marques OAV, Sazima I, Schuett GW, Hoggren M, Douglas ME (2002). Ecological and phylogenetic correlates of feeding habits in Neotropical pitvipers of the genus *Bothrops*. Biology of the vipers.

[ref-42] McDowell SB (1974). A catalogue of the snakes of New Guinea and the Solomons, with special reference to those in the Bernice P. Bishop Museum, Part l. Scolecophidia. Journal of Herpetology.

[ref-43] Miller KL, Castañeda Rico S, Muletz-Wolz CR, Campana MG, McInerney N, Augustine L, Fleischer RC (2019). Parthenogenesis in a captive Asian water dragon (*Physignathus cocincinus*) identified with novel microsatellites. PLOS ONE.

[ref-44] Neaves WB, Baumann P (2011). Unisexual reproduction among vertebrates. Trends in Genetics.

[ref-45] Nogueira CC, Argôlo AJ, Arzamendia V, Azevedo JA,  Barbo FE,  Bérnils RS,  Bolochio BE, Borges-Martins  M, Brasil-Godinho M, Braz H (2019). Atlas of Brazilian snakes: verified point-locality maps to mitigate the Wallacean shortfall in a megadiverse snake fauna. South American Journal of Herpetology.

[ref-46] Nussbaum RA (1980). The brahminy blind snake (*Ramphotyphlops braminus*) in the Seychelles archipelago: distribution, variation, and further evidence for parthenogenesis. Herpetologica.

[ref-47] Olsen MW (1967). Age as a factor influencing the level of parthenogenesis in eggs of turkeys. Experimental Biology and Medicine.

[ref-48] Olsen MW (1970). Weights of some internal organs and glands of fully developed parthenogenetic and normal turkey embryos. Poultry Science.

[ref-49] Olsen MW (1975). Avian parthenogenesis. Agricultural Research Service USDA, ARS-NE.

[ref-50] Ota H, Hikida T, Matsui M, Mori A, Wynn AH (1991). Morphological variation, karyotype and reproduction of the parthenogenetic blind snake, Ramphotyphlops braminus, from the insular region of East Asia and Saipan. Amphibia-Reptilia.

[ref-51] Parker HM, McDaniel CD (2009). Parthenogenesis in unfertilized eggs of *Coturnix chinensis*, the Chinese painted quail, and the effect of egg clutch position on embryonic development. Poultry Science.

[ref-52] Pokorná M, Rens W, Rovatsos M, Kratochvíıl L (2014). A ZZ/ZW sex chromosome system in the thick-tailed gecko (*Underwoodisaurus milii*; Squamata: Gekkota: Arphodactylidae), a member of the ancient gecko lineage. Cytogenetic and Genome Research.

[ref-53] Portnoy DS, Hollenbeck CM, Johnston JS, Casman HM, Gold JR (2014). Parthenogenesis in a whitetip reed shark *Triaenodon obesus* involves a reduction in ploidy. Journal of Fish Biology.

[ref-54] Ramachandran R, McDaniel CD (2018). Parthenogenesis in birds: a review. Reproduction.

[ref-55] Robinson DP, Baverstock W, Al-Jaru A, Hyland K, Khazanehdari KA (2011). Annually recurring parthenogenesis in a zebra shark *Stegostoma fasciatum*. Journal of Fish Biology.

[ref-56] Rovatsos M, Vukíc J, Lymberakis P, Kratochvíl L (2015). Evolutionary stability of sex chromosomes in snakes. Proceedings of the Royal Society B.

[ref-57] Schuett GW, Campbell JA, Brodie Jr ED (1992). Is long-term sperm storage an important component of the reproductive biology of temperate pitvipers?. Biology of the pitvipers.

[ref-58] Schut E, Hemmings N, Birkhead TR (2008). Parthenogenesis in a passerine bird, the zebra finch *Taeniopygia guttata*. Ibis.

[ref-59] Seixas F, Morinha F, Luis C, Alvura N, Dos Anjos-Pires M (2020). DNA-validated parthenogenesis: first case in a captive Cuban boa (*Chilabothrus angulifer*). Salamandra, German Journal of Herpetology.

[ref-60] Senzaki BM (2020). Caracterização citogenética de serpentes em um contexto filogenético. Course Conclusion Monography.

[ref-61] Shibata H, Sakata S, Hirano Y, Nitasaka E, Sakabe A (2017). Facultative parthenogenesis validated by DNA analyses in the green anaconda (*Eunectes murinus*). PLOS ONE.

[ref-62] Siegel DS, Sever DM (2006). Utero-muscular twisting and sperm storage in viperids. Herpetological Conservation and Biology.

[ref-63] Silva KMP, Silva KB, Sueiro LR, Oliveira MES, Almeida-Santos SM (2019). Reproductive biology of Bothrops atrox (Serpentes, Viperidae, Crotalinae) from the Brazilian Amazon. Herpetologica.

[ref-64] Smith CF, Schuett GW, Earley RL, Schwenk K (2009). The spatial and reproductive ecology of the copperhead (*Agkistrodon contortrix*) at the northeastern extreme of its range. Herpetological Monographs.

[ref-65] Smith CF, Schuett GW, Schwenk K (2010). Relationship of plasma sex steroids to the mating season of copperheads at the northeastern extreme of their range. Journal of Zoology.

[ref-66] Stenberg P, Saura A, Schön I, Martens K, Van Dijk P (2009). Cytology of asexual animals. Lost sex: the evolutionary biology of parthenogenesis.

[ref-67] Straube N, Lampert KP, Geiger MF, Weiß JD, Kirchhauser JX (2016). First record of second-generation facultative parthenogenesis in a vertebrate species, the whitespotted bambooshark *Choloscyllium plagiosum*. Journal of Fish Biology.

[ref-68] Vaughan MS, Steele RA (2014). *Bothrops* asper (Terciopelo). Parthenogenetic reproduction. Natural History Notes. Herpetological Review.

[ref-69] Vrijenhoek RC, Knobil E, Neill JD (1999). Parthenogenesis and natural clones. Encyclopedia of Reproduction.

[ref-70] Walsh PS, Metzger DA, Higuchi R (1991). Chelex 100 as a medium for simple extraction of DNA for PCR-based typing from forensic material. Biotechniques.

[ref-71] Watts PC, Buley KR, Sanderson S, Boardman W, Ciofi C, Gibson R (2006). Parthenogenesis in Komodo dragons. Nature.

[ref-72] Wiechmann R (2012). Observations on parthenogenesis in monitor lizards. Biawak.

[ref-73] Wüster W, Thorpe RS, Puorto G, BBBSP (1996). Systematics of the *Bothrops atrox* complex (Reptilia: Serpentes: Viperidae) in Brazil: a multivariate analysis. Herpetologica.

[ref-74] Wynn AH, Cole CJ, Gardner AL (1987). Apparent triploidy in the unisexual brahminy blind snake, Ramphotyphlops braminus. American Museum Novitates.

