# Supplementary figures and images for: Evidence of facultative parthenogenesis in three Neotropical pitviper species of the Bothrops atrox group
Source: PeerJ. 2020 Nov 18;8:e10097. doi: 10.7717/peerj.10097 (PMC7680053; doi:10.7717/peerj.10097)

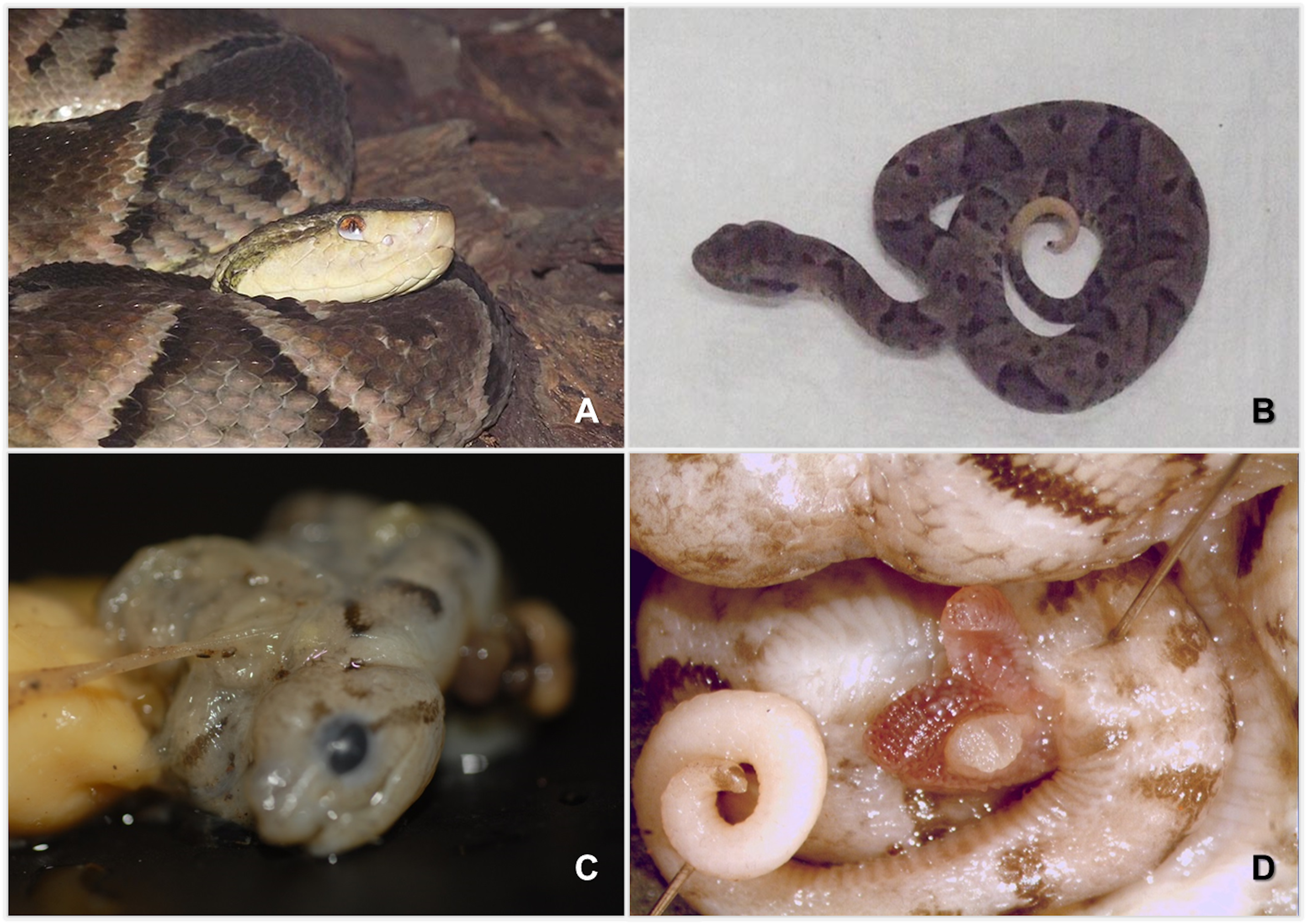

Supplement: Figure S1 — (A) Mother BUT44. (B) Offspring of mother BUT44. (C) Malformed embryo—attached to de yolk—of mother BUT44. (D) Hemipenis of the embryo of mother BUT44. [file peerj-08-10097-s001.png]

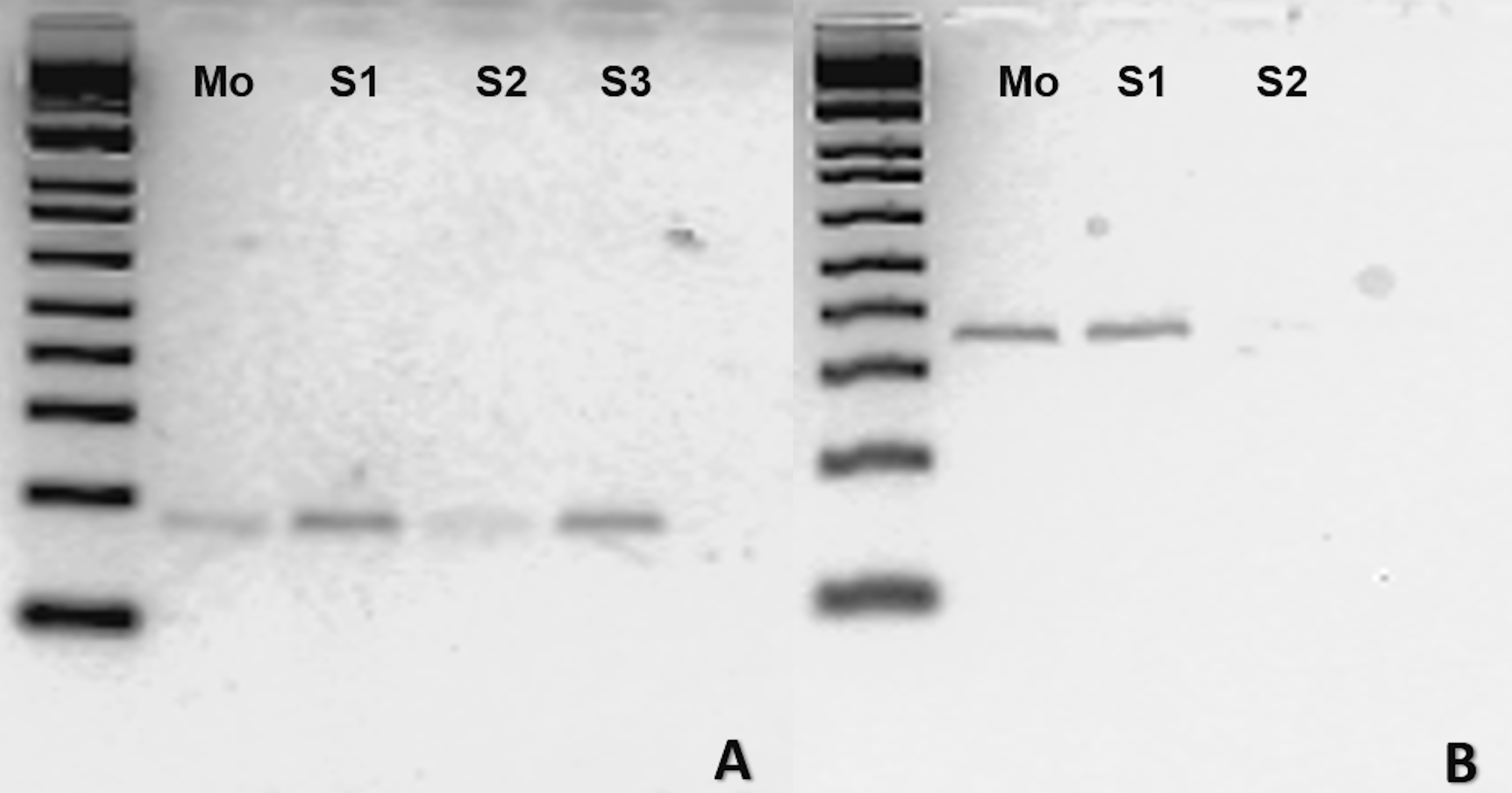

Supplement: Figure S2 — (A–B), where Mo: putative parthenogenetic mother; S1–S3; (C-): negative control. The first lane in all figures is a molecular weight standard. Loci: (A) MR102. (C) Bi52.13. [file peerj-08-10097-s002.png]

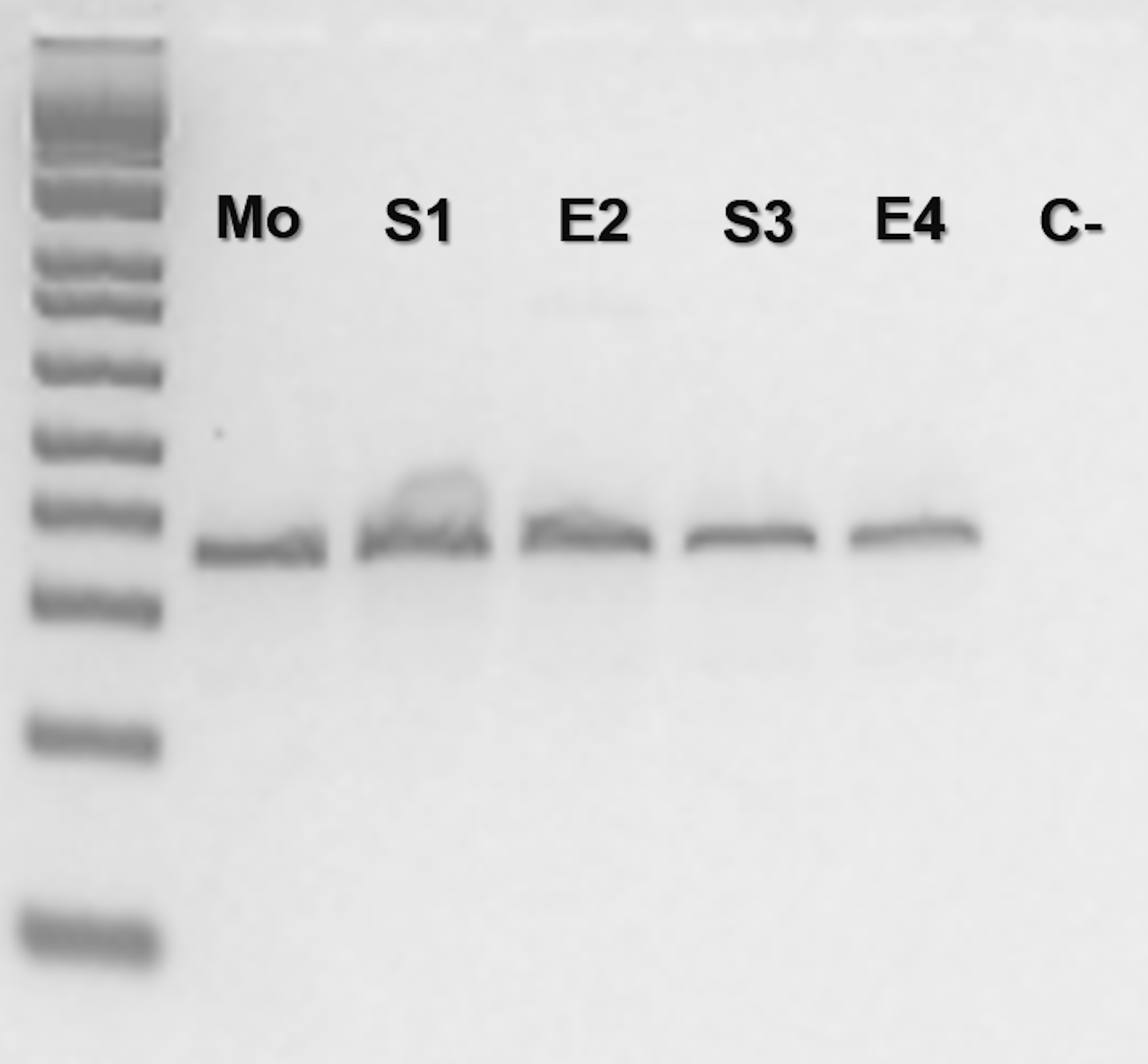

Supplement: Figure S3 — Where Mo: putative parthenogenetic mother; S1, E2, S3, and S4: sons of Mo (S1 and E2 were born in 2016, and S3 and E4 were born in 2018) and (C-): negative control. In the first lane: the molecular Low Mass Ladder 1kb. [file peerj-08-10097-s003.png]
