# Supplementary material for: Evidence of facultative parthenogenesis in three Neotropical pitviper species of the Bothrops atrox group
Source: PeerJ. 2020 Nov 18;8:e10097. doi: 10.7717/peerj.10097 (PMC7680053; doi:10.7717/peerj.10097)
Supplement: Table S1 — Loci from which informative PCR bands of three out of four suspected Bothrops facultative parthenogenesis cases were obtained, depicted by species. Numbers refer to estimated gel band sizes. [file peerj-08-10097-s004.docx]

**Table S1:**

Loci from which informative PCR bands of three out of four suspected *Bothrops* facultative parthenogenesis cases were obtained, depicted by species. Numbers refer to estimated gel band sizes

| **Species**  **Mother/Offspring** | **Locus/ Allele size**   \| **Ac4335** \| **MR102** \| **Bi52.13** \| **Bi60.3** \| \| --- \| --- \| --- \| --- \| | | | |
| --- | --- | --- | --- | --- | --- | --- | --- | --- |
| ***B. atrox*** |  |  |  |  |
| Mo (ID#933) | 280/266 | 188/163 | 350/350 | 368/355 |
| S1 | 266/266 | 163/163 | 350/350 | 368/368 |
| S2 |  | 188/188 | 350/350 | - |
| ***B. moojeni*** |  |  |  |  |
| Mo (BUT44) | 215/215 | 188/188 | - | 380/380 |
| S1 | 215/215 | 188/188 | - | 380/380 |
| S2 | 215/215 | 188/188 | - | 380/380 |
| S3 | 215/215 | 188/188 | - |  |
| ***B. moojeni*** |  |  |  |  |
| Mo (BUT86) | 289/215 | 187/172 | 617/617 | 356/356 |
| S1 | 215/215 | 187/187 | 617/617 | 356/356 |
| E2 | 289/289 | 187/187 | 617/617 | 356/356 |
| O1 | - | 187/187 | - | - |
| O2 | - | 187/187 | - | - |
| O3 | - | 172/172 | - | - |
| S3 | 289/289 | 172/172 | 617/617 | 356/356 |
| E4 | 215/215 | 172/172 | 617/617 | 356/356 |
| O4 | - | 172/172 | - | - |
| O5 | - | 172/172 | - | - |
| O6 | - | 187/187 | - | - |
| ***B. leucurus*** |  |  |  |  |
| Mo (MJJS503) | 265/225 | 195/177 | 645/512 | 334/334 |
| E1 | 225/225 | 177/177 | 512/512 | 334/334 |
| O1 | - | 177/177 | 512/512 | 334/334 |
