# Supplementary material for: Evidence of facultative parthenogenesis in three Neotropical pitviper species of the Bothrops atrox group
Source: PeerJ. 2020 Nov 18;8:e10097. doi: 10.7717/peerj.10097 (PMC7680053; doi:10.7717/peerj.10097)
Supplement: Table S2 — “Autosome composition” assumes that the mother is heterozygous in a specific locus. Crossing-over, as depicted here, is between the centromere and the locus. [file peerj-08-10097-s005.docx]

**Table S2:**

**Known cytogenetic mechanisms involved in animal parthenogenesis (compiled from Stenberg & Saura, 2009). “Autosome composition” assumes that the mother is heterozygous in a specific locus. Crossing-over, as depicted here, is between the centromere and the locus.**

|  | **Sex composition** | **Autosome composition** | **Crossing-over** | **No crossing** |
| --- | --- | --- | --- | --- |
| **Automixis** |  |  |  |  |
| Gamete duplication | ZZ / WW | 100% homozygosity | AA / aa | AA / aa |
| Terminal fusion | ZZ / WW^1^ | AA / Aa / aa | Aa | AA / aa |
| Central fusion | ZZ / ZW / WW | AA / Aa / aa | AA / Aa / aa | Aa |
| 1st polar + secondary oocite | ZZ / ZW / WW^2^ | AA / Aa / aa^2^ | AA / Aa / aa^2^ | AA / Aa / aa^2^ |
| Gonoid thelytoky | ZW | same as mother | - | same as mother |
| Premeiotic doubling | ZW | same as mother | same as mother | same as mother |
| **Apomixis** | ZW | same as mother | - | - |

^1^ ZW theoretically possible if sexual chromosomes cross-over during meiosis I.

^2^ Depending on the details, offspring is genetically identical to the mother.
